# Quercetin mitigates aluminum nanoparticle-induced neurotoxicity: a stereological and molecular study on memory, hippocampal integrity, and MAPK signaling

**DOI:** 10.17179/excli2025-8315

**Published:** 2025-07-02

**Authors:** Zahra Esmaili, Mohammad Shabani, Fatemeh Karimi, Moazamehosadat Razavinasab, Meysam Ahmadi-Zeidabadi, Majid Reza Farokhi, Maryam Moosavi

**Affiliations:** 1 Neuroscience Research Center, Institute of Neuropharmacology, Kerman University of Medical Sciences, Kerman, Iran; 2 Histomorphometry and Stereology Research Center, Shiraz University of Medical Sciences, Shiraz, Iran; 3 Department of Physiology and Pharmacology, School of Medicine, Kerman University of Medical Sciences, Kerman, Iran; 4 Shiraz Neuroscience Research Centre, Shiraz University of Medical sciences, Shiraz, Iran; 5 Nanomedicine and Nanobiology Research Center, Shiraz University of Medical Sciences, Shiraz, Iran

**Keywords:** aluminum nanoparticles, quercetin, hippocampus, Alzheimer's disease, MAPK signaling, stereology, neuroprotection

## Abstract

Emerging evidence suggests a strong association between aluminum (Al) exposure and the development of Alzheimer's disease (AD). Due to their nanoscale size and increased surface area, Al nanoparticles (ALNP) exhibit greater neurotoxicity than bulk Al, raising concerns about their role in neurodegenerative disorders. While quercetin has been recognized for its neuroprotective effects, its ability to counteract ALNP-induced hippocampal neurodegeneration and dysregulated MAPK signaling remains largely unexplored. This study investigated the potential of quercetin to ameliorate ALNP-induced memory deficits, alterations in hippocampal stereological parameters, and disruptions in caspase-3 and MAPK signaling in male Swiss mice. Mice (SWR/J, aged 8-10 weeks) received ALNP (10 mg/kg, intraperitoneally for 10 days) with or without quercetin at doses of 1, 10, or 100 mg/kg (orally). Memory performance was assessed using the elevated plus maze (EPM), novel object recognition (NOR), and Y-maze tasks, followed by stereological and western blot analyses of the hippocampus. Our findings revealed that quercetin (100 mg/kg) significantly preserved hippocampal volume and neuronal integrity in the dentate gyrus (DG) and Cornu Ammonis 1 (CA1)-key regions involved in memory processing and output signaling. Additionally, quercetin modulated MAPK signaling by enhancing ERK phosphorylation while suppressing ALNP-induced activation of p38 and cleaved caspase-3, suggesting a role in reducing neuroinflammation and apoptosis. This is the first study to demonstrate that quercetin can counteract the neurotoxic effects of ALNP, highlighting its potential as a therapeutic strategy against nanoparticle-induced neurodegeneration in an Alzheimer's-like model.

See also the graphical abstract.(Fig. 1)

## Introduction

Aluminum (Al) exposure has been implicated in the pathogenesis of Alzheimer's disease (AD), with postmortem analyses revealing elevated Al levels in the hippocampus and temporal cortex of AD patients (Lukiw et al., 2019[43]; Rusina et al., 2011[58]). The neurotoxicity of Al is largely attributed to Al³⁺ ions, which can promote the aggregation of amyloid-beta (Aβ) and hyperphosphorylated tau, impair cholinergic neurotransmission, and induce oxidative stress and inflammation, all of which are key hallmarks of AD pathology (Dey and Singh, 2022[17]). Accordingly, many experimental models of AD use Al salts such as AlCl₃ to mimic these neuropathological changes. However, increasing attention has been paid to Al-based nanomaterials, particularly Al oxide nanoparticles (ALNP), due to their widespread application and potential neurotoxicity (Betzer et al., 2017[10]; Shilo et al., 2014[62]; Zhou et al., 2018[73]). Unlike ionic Al, ALNP are largely insoluble under physiological conditions and exhibit distinct physicochemical properties that enable them to cross biological barriers, induce oxidative stress, and alter intracellular signaling pathways (Avramescu et al., 2022[8]; Shah et al., 2015[60]; Zhou et al., 2018[74]). Compared to ionic forms, these nanoparticles exhibit greater surface reactivity and pro-oxidant capacity, potentially leading to heightened neurotoxicity (Abd-Elhakim et al., 2021[1]). Recent in vivo studies have demonstrated that exposure to ALNP can result in memory impairment, hippocampal atrophy, and modulation of MAPK signaling- supporting their role as potent neurotoxicants (Esmaili et al., 2022[19]; Mehrbeheshti et al., 2022[44]; Shah et al., 2015[60]). These nanoparticle-specific effects merit investigation, as they may induce neurodegeneration through mechanisms that are independent of free Al³⁺ ion release.

The hippocampus, a critical structure for learning and memory, is composed of the dentate gyrus (DG) and Cornu Ammonis (CA) subfields, with DG serving as the primary input gateway and CA1 functioning as a key output station (Rao et al., 2022[55]). Structural and cellular damage within these regions correlates strongly with cognitive decline in AD (Kanagamani et al., 2023[33]; Rao et al., 2022[55]). At the molecular level, hippocampal neurodegeneration is closely linked to dysregulation of the mitogen-activated protein kinase (MAPK) pathway, which includes p38 MAPK, c-Jun N-terminal kinase (JNK), and extracellular signal-regulated kinase (ERK). While ERK activation is associated with neuroprotection and synaptic plasticity, excessive p38 and JNK activation contribute to inflammation, apoptosis, and neurodegeneration (Kheiri et al., 2018[34]; Kim and Choi, 2015[35]; Yarza et al., 2015[68]).

Given the limited efficacy and side effects of current AD pharmacotherapies, interest in natural neuroprotective agents has surged (Hussain and Bloemer, 2023[27]). Quercetin, a flavonoid with potent antioxidant and anti-inflammatory properties, has shown promise in mitigating AD-related pathology, including memory impairment and neuronal loss (Nishihira et al., 2021[49]; Sabogal-Guáqueta et al., 2015[59]). Previous studies have reported that quercetin protects against Al-induced neurotoxicity, but its effects against ALNP-induced hippocampal degeneration and MAPK dysregulation remain largely unexplored (Alaqeel et al., 2022[3]; Jadhav and Kulkarni, 2023[31]). Notably, due to its nanoscale properties, ALNP may exert distinct toxic effects compared to bulk Al, necessitating targeted neuroprotective interventions.

This study aimed to evaluate the neuroprotective effects of quercetin against ALNP-induced hippocampal damage using an advanced stereological approach, a technique that provides precise, unbiased estimations of hippocampal volume and neuronal counts, factors crucial for assessing neurodegeneration. Furthermore, we investigated quercetin impact on MAPK signaling, with a focus on ERK, p38, JNK, and caspase-3 activation, to delineate its potential mechanisms of neuroprotection. By addressing a critical gap in the literature, this study provides novel insights into quercetin role in mitigating nanoparticle-induced neurotoxicity and highlights its therapeutic potential for AD-related neurodegeneration.

## Material and Methods

### Animals and ethics statement

Adult male Swiss mice (SWR/J, aged 8-10 weeks) were obtained from the animal facility of Shiraz University of Medical Sciences, Shiraz, Iran. The animals were housed in a controlled environment with a temperature of 24 ± 1 °C and a relative humidity of 55 ± 5%, with water and food *ad libitum*. All studies were performed according to with the ARRIVE principles and the National Institutes of Health Guide for the Care and Use of Laboratory Animals (NIH Publication No. 80-23, updated 1996). The Ethics Committee for Scientific Study at Kerman University of Medical Sciences has approved this work under the reference number IR. KMU.AH.REC.1402.046. 

### Chemicals

Aluminum oxide nanoparticles (ALNP, Sigma-Aldrich, #544833, St. Louis, MO, USA) were used in this study as a dry powder. The characteristics of the product include gamma-phase alumina NPs with a particle size of less than 50 nm and a surface area of 40 m^2^/g (measured using the Brunauer-Emmett-Teller method). ALNP was dissolved in a normal saline solution. Quercetin was procured from Sigma (#Q4951, St. Louis, MO, United States) at a purity exceeding 95% as determined by high performance liquid chromatography. The Western blot antibodies consisted of phosphorylated-p38 (p-p38, Rabbit monoclonal antibody (mAb), #4511), total p38 (t-p38, Rabbit mAb, #8690), phosphorylated-JNK (p-JNK, Rabbit mAb, #4671), total JNK (t-JNK, Rabbit mAb, #9252), phosphorylated-ERK (p-ERK, Rabbit mAb, #43771), total ERK (t-ERK, Rabbit mAb, #4695), caspase-3 (Rabbit mAb, #9665), and Anti-Rabbit IgG, horseradish-peroxidase (HRP)-linked antibody (#7074), were sourced from Cell Signaling Technology, Danvers, MA, USA. The GAPDH (Glyceraldehyde 3-phosphate dehydrogenase) antibody (mouse mAb, #sc-166574) was acquired from Santa Cruz Biotechnology, Dallas, TX, USA. Additionally, the anti-mouse HRP-linked antibody (#554002) was obtained from the BD PharMingen, San Diego, USA. The Halt protease and phosphatase inhibitor cocktail was provided by Thermo Scientific (#78445); the Amersham ECL select reagent kit (#RPN2235) was procured from GE Healthcare Life Sciences, UK, and the PVDF membrane (#IPVH00010) was sourced from Millipore. All other reagents were purchased from typical commercial suppliers.

### Drug administration and experimental design

**Figure 2:**
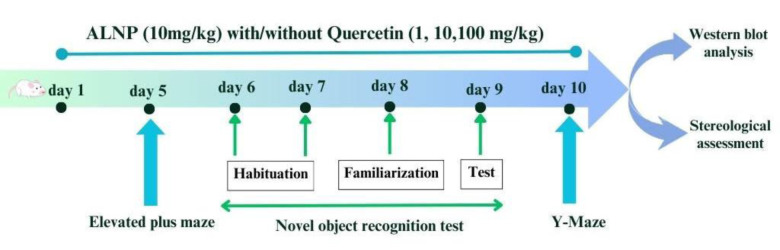
The timeline of behavioral, stereological, and western blot experiments

The study included 40 mice, organized into five groups of eight each over a 10-day period. ALNP (10 mg/kg, intraperitoneally (i.p.) was suspended in saline and sonicated for 30 minutes prior to administration. Quercetin was suspended in 2% carboxymethyl cellulose (CMC) and administered in doses of 1, 10, and 100 mg/kg orally. Quercetin and ALNP were administered 60 and 30 minutes, respectively, before each behavioral session (Fig. 2(Fig. 2)). The doses of ALNP (Esmaili et al., 2022[19]; Mehrbeheshti et al., 2022[44]) and quercetin (Paula et al., 2019[51]; Rishitha and Muthuraman, 2018[56]) were selected based on previous studies. The experimental groups were as follows:

1. Control group: Mice in this group received isotonic saline (i.p.) and carboxymethyl cellulose (CMC 2%) (oral gavage) as the vehicle of ALNP and quercetin, respectively.
2. ALNP group: Mice in this group received CMC 2% (oral gavage) and ALNP (10 mg/kg/ i.p.).
3. ALNP+Q1 group: Mice in this group received ALNP (10 mg/kg/ i.p.) and quercetin 1 mg/kg (oral gavage).
4. ALNP+Q10 group: Mice in this group received ALNP (10 mg/kg/ i.p.) and quercetin 10 mg/kg (oral gavage) and ALNP (i.p.).
5. ALNP+Q100 group: Mice in this group received ALNP (10 mg/kg/ i.p.) + quercetin 100 mg/kg (oral gavage).

### Behavioral assessments

#### Experiment 1: Elevated plus maze test (EPM)

The elevated plus maze test was conducted on the fifth day of the experiment to assess anxiety-like behaviors in the experimental groups. The maze, elevated 45 cm above the floor, consisted of two open (30 × 5 × 0.2 cm) and two closed (30 × 5 × 15 cm) arms extending from a central platform (5 × 5 cm). Animals were placed in the maze for 5 minutes, positioned in the center towards an open arm. The number of entries into both types of arms were recorded (Javurek et al., 2017[32]). Arm entries were defined as the animal placing its forepaws into an arm. The total number of close arm entries was used as measure of general activity (Walf and Frye, 2007[66]).

#### Experiment 2: Novel object recognition test (NOR)

The Novel Object Recognition (NOR) test was employed to evaluate cognitive ability in mice (Lueptow, 2017[42]). The NOR apparatus consisted of a Plexiglas open field arena (40 × 40 cm). Mice were allowed to acclimate to the empty arena over two sessions, each lasting 5 minutes, with a 1-hour interval between sessions across two days (Mehrbeheshti et al., 2022[44]). On the eighth day of the experiment, a familiarization session was conducted in which two identical objects (A1 and A2) were placed in the arena, each positioned within 5 cm of the wall. The mice were introduced to the arena facing away from the objects. Exploration was defined as the use of the nose or forepaws to sniff or touch the objects. The familiarization session lasted 10 minutes, and mice that did not explore for a minimum duration of 20 seconds were excluded from the study (Lueptow, 2017[42]). Twenty-four hours later, during the test trial, which also lasted 10 minutes, one familiar object (A1) and one novel object (B) were presented. The objects and arena were thoroughly cleaned with a 70% ethanol solution after each trial. The Discrimination Index (DI) was calculated using established formulas:

DI = (Exploration time of novel object - Exploration time of familiar object) / Total exploration time.

#### Experiment 3: Y-maze test

The Y-maze test was employed to evaluate reference spatial memory (Kraeuter et al., 2019[36]). The maze consisted of a Y-shaped compartment made of Plexiglass material, featuring equal-length arms measuring 21 × 7 × 15.5 cm. The Plexiglas apparatus had three perpendicular arms labeled A, B, and C, with a 120° angle between each arm. Three distinct visual cues were positioned around the maze arms. The test comprised two trials with an inter-trial interval of one hour (Kraeuter et al., 2019[36]). During the training trial, the mice were allowed to explore the A (starting arm) and B arms for fifteen minutes, while in the test trial, all three arms were accessible for five minutes (Kraeuter et al., 2019[36]). The walls and floor of the maze were cleaned with 70% ethanol before proceeding to the next animal. The percentage of time spent in the novel arm (C) and the proportion of entries into the novel arm were measured for each mouse. Since the mice tend to remain in the start arm (A) during the initial seconds of the trials, this arm was excluded from the comparison, and only arms B and C were compared (Kraeuter et al., 2019[36]).

### Stereological assessment

The stereological methods measured key quantitative parameters, including the total volume of the hippocampus and its CA1 and DG sub-regions, as well as the neural cell counts in the CA1 and DG regions.

#### Tissue preparation

**Figure 3:**
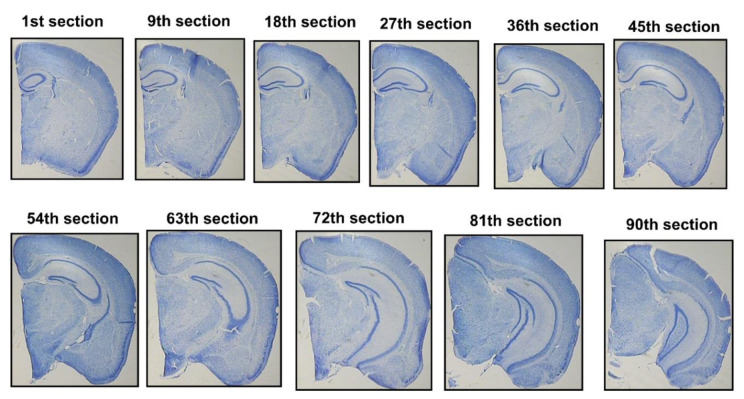
Coronal serial sections of a mouse brain stained with Giemsa.

Following the behavioral tests, five mice per group were randomly selected (Zhang et al., 2020[70]). Considering that quercetin at a dosage of 100 mg/kg effectively mitigated memory impairment in both the NOR and Y-maze tasks, this group was selected for further stereological studies. The animals were anesthetized with ketamine (100 mg/kg) and xylazine (10 mg/kg). Next, a transcardial infusion of saline solution (0.9%) was performed until the liver exhibited complete blood clearance. Subsequently, buffered formalin (10%) was infused to fix the brain tissue. After completing the perfusion method, the brain was extracted and post-fixed in a 10% formalin solution. The paraffin-embedded brain blocks were sectioned into 25 µm coronal slices and stained using the modified Giemsa procedure (Iñiguez et al., 1985[28]). Using systematic random sampling, 10 to 11 brain sections (Fig. 3(Fig. 3)) were selected from each mouse (Long et al., 1998[40]). 

#### Estimations of the volumes of the entire hippocampus, CA1, and dentate gyrus sub-regions

**Figure 4:**
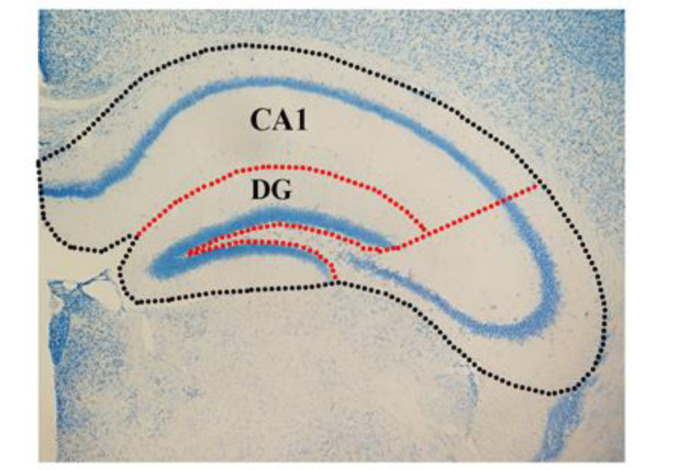
Figure 4: The coronal section of the hippocampal formation (4x) showing the contours of the entire hippocampus (black dotted line), Cornu ammonis1 (CA1) and dentate gyrus DG (red dotted line).

Using a 4x microscope, the entire hippocampal tissue was scanned, and the contours of the whole hippocampus, as well as the CA1 and DG subfields (Fig. 4(Fig. 4)), were delineated using the atlas of Paxinos and Watson (Paxinos and Franklin, 2012[52]). The Cavalieri technique was employed to determine the volume of the entire hippocampus and its sub-regions, DG and CA1 (Kristiansen and Nyengaard, 2012[37]). To achieve this, a point grid was superimposed on the images of each section using stereology software developed by Shiraz University of Medical Sciences. The volumes of the entire hippocampus, as well as the CA1 and DG sub-regions, were calculated by estimating the number of points that intersected with the sampled sections (∑P) and multiplying this sum by the area associated with each point (a/p) and the distance between the sections (d). Ultimately, the volume was approximated using the following formula: V = ∑P × (a/p) × d.

#### Estimations of the neuronal number of CA1 (pyramidal) and DG (granular) neural cells

The total number of the neural cells in the CA1 and DG was determined using a light microscope (Nikon E200, Japan) with an oil immersion lens (40X, numerical aperture = 1.3) connected to a computer system. The optical dissector approach involved overlaying an unbiased counting frame over brain section pictures with accepted (top and right) and banned borders (bottom and left). Microscopic fields were investigated by moving the microscope stage in both X and Y directions at equal intervals to provide a consistent and random sampling process (Gundersen et al., 1988[23]; Kristiansen and Nyengaard, 2012[37]). Nuclei of the cells that were completely or partially positioned inside the counting frame and did not touch the left and bottom borders were selected. The numerical density (Nv) in CA1 and DG was multiplied by V (CA1 or DG) to estimate the total number of cells. The formula for estimating the total number of cells was Nv (cells / CA1 or DG) = [∑Q/∑P × (a/f) × h] × [t/BA], where "ΣQ−" represents the total number of nuclei in CA1 and DG that came into focus during scanning, "ΣP" refers to the total number of counting frames in most fields, "h" is the height of the dissector, "a/f" is the frame area, "t" is the mean section thickness determined in each sampled field using the microcator (20 μm on average), and "BA" is the block advance of the microtome set at 25 μm (Rubinow and Juraska, 2009[57])

#### Three-dimensional reconstruction (3DR)

To produce a three-dimensional reconstruction (3DR) of the hippocampal structure. The tissue section photographs were prepared using Z-stacks at a magnification of 25x. To assess the three-dimensional characteristics of the hippocampus, CA1 and DG, a 3DR representation of the structures was generated using the "RECONSTRUCT" program (http://synapses.clm.utexas.edu). This open-source program facilitates the creation of 3D figures by effectively recreating the montage, alignment, and display of serial portions (Helander, 2006[26]).

### Western blot analysis

Following the behavioral tests, three mice per group were randomly selected for western blot studies. Considering that quercetin at a dosage of 100 mg/kg effectively mitigated memory impairment in both the NOR and Y-maze tasks, this group was selected for further western blot studies. The animals were euthanized using CO2 inhalation. After decapitation using the guillotine method, the brains were removed, and the hippocampi were isolated on ice, snap-frozen in liquid nitrogen, and stored at -80°C for molecular assays. The tissues were lysed using cold Radioimmunoprecipitation Assay (RIPA) lysis buffer, which included protease and phosphatase inhibitors. After centrifugation at 12,000 rpm for 20 minutes at 4°C, the supernatant was collected, and the protein content was measured using the modified Lowry method (Hartree, 1972[25]; Lowry et al., 1951[41]). In summary, equal amounts of protein from each sample (50 μg) were loaded onto 10% SDS-polyacrylamide gels, which included molecular weight markers (Askari et al., 2022[6]). The separated proteins were then transferred to polyvinylidene difluoride (PVDF) membranes at 4°C using a cold pack and pre-chilled buffer. After blocking with 5% bovine serum albumin in TBST (Tris-buffered saline with Tween-20), the membranes were probed with primary antibodies overnight at 4°C. The membranes were washed three times with TBST for 10 minutes each and subsequently incubated with horseradish peroxidase-conjugated anti-rabbit antibodies for 2 hours. The membranes were then washed three additional times, and band detection was performed using the ECL kit. The signals on the developed films were quantified using ImageJ software. Stripping and re-probing were conducted to assess the levels of phosphorylated proteins before measuring the total levels of the same proteins (Bass et al., 2017[9])

### Statistical analysis 

GraphPad Prism 10 was utilized for statistical analysis. The normal distribution of data was assessed using the Kolomogrov-Smirnov test. For normally-distributed data, a one-way ANOVA followed by Tukey's post-hoc test was used for multiple comparisons between groups. Pearson's correlation analysis was employed to investigate the potential associations between neural cell number and the volumes of the CA1 and DG sub-regions. Results are presented as mean ± SEM. P<0.05 was considered statistically significant. 

## Results

### The Effects of quercetin on anxiety-like behaviors in the EPM test in ALNP treated mice

The behavioral results of the elevated plus maze (EPM) test are presented in Table 1(Tab. 1). Columns 1 and 2 represent the percentage of entries into the open and closed arms, respectively. No significant differences were observed between groups. Column 3 displays the number of entries into the closed arms, which serves as a measure of general activity (Walf and Frye, 2007[66]) with no significant difference between groups. These results suggest that ALNP treatment, whether administered alone or with quercetin, does not impact the anxiety levels of the animals.

### Quercetin prevents the impairing effect of ALNP on novel object recognition (NOR) memory deficit

**Figure 5:**
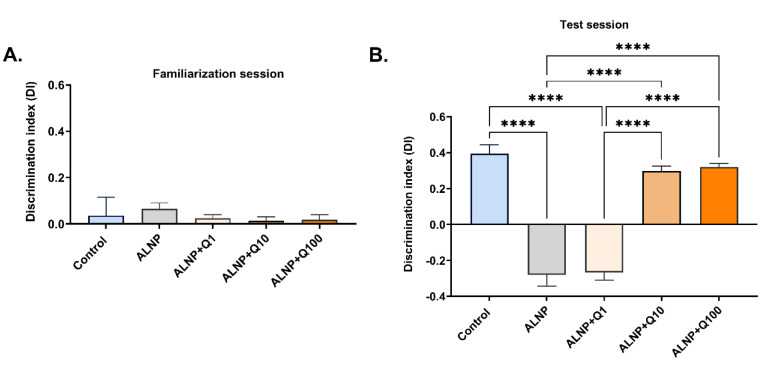
The effect of ALNP with or without quercetin on the performance in the novel object recognition (NOR) test in mice. The discrimination index (DI) for object A2 during the familiarization session (Fig. 5A) and the DI for the unfamiliar object (object B) during the test session (Fig. 5B) are presented. The data are expressed as the mean ± SEM (n = 8 per group). (****P < 0.0001) indicate significant differences between groups. The groups include: control (saline + CMC 2%), ALNP (aluminum nanoparticles 10 mg/kg + CMC 2%), ALNP + Q1 (aluminum nanoparticles 10 mg/kg + quercetin 1 mg/kg), ALNP + Q10 (aluminum nanoparticles 10 mg/kg + quercetin 10 mg/kg), and ALNP + Q100 (aluminum nanoparticles 10 mg/kg + quercetin 100 mg/kg).

Figure 5(Fig. 5) illustrates the behavioral results of the novel object recognition (NOR) test. During the familiarization session (Fig. 5A(Fig. 5)), no significant differences were observed in the discrimination index (DI) across groups (F (4, 35) = 0.9230, P > 0.05). In the test session, a one-way ANOVA revealed significant differences in the DI (Fig. 5B(Fig. 5), F (4, 35) = 61.33, P < 0.0001). Post hoc Tukey analysis indicated that ALNP significantly decreased the DI, while the quercetin-treated groups at doses of 10 and 100 mg/kg did not exhibit such a decrease and significantly differed from the ALNP group. No significant difference was observed in the DI between the ALNP+Q1 group and the ALNP group during test session. These results indicate that ALNP negatively affects cognitive memory, whereas quercetin at doses of 10 and 100 mg/kg reduces this effect. 

### Quercetin prevents the detrimental effects of ALNP on reference spatial memory in the Y-maze test

**Figure 6:**
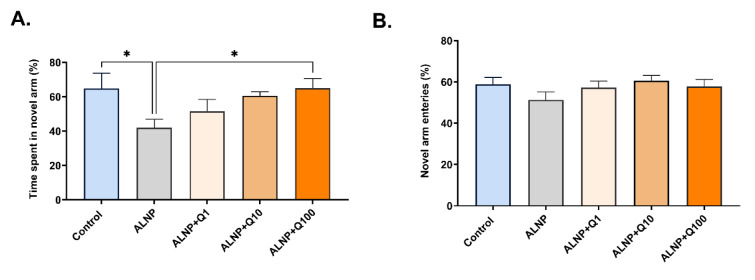
Figure 6. Effect of ALNP with or without quercetin on Y-maze spatial reference memory. The percentage of time (A) and entries (B) spent in the novel arm during the Y-maze test. The data are expressed as mean ± SEM (n = 8 for each group). *P < 0.05 shows the difference between groups. The groups include: control (saline + CMC 2%), ALNP (aluminum nanoparticles 10 mg/kg + CMC 2%), ALNP + Q1 (aluminum nanoparticles 10 mg/kg + quercetin 1 mg/kg), ALNP + Q10 (aluminum nanoparticles 10 mg/kg + quercetin 10 mg/kg), and ALNP + Q100 (aluminum nanoparticles 10 mg/kg + quercetin 100 mg/kg).

Y-maze test was conducted to assess reference spatial short-term memory. Figure 6A(Fig. 6) illustrates the percentage of time spent in the novel arm of the Y-maze during the test session. A one-way ANOVA revealed a significant difference between groups (F (4, 35) = 4.139, P = 0.0075). The ALNP + Q100 mg/kg group spent more time in the novel arms compared to the ALNP group (Fig. 6A(Fig. 6)). Although quercetin at doses of 1 and 10 mg/kg appeared to increase the percentage of time spent in the novel arm, no statistically significant differences were observed compared to the ALNP group. Figure 6B(Fig. 6) illustrates the percentage of entries into the novel arm during the Y-maze test session. A one-way ANOVA revealed no significant differences between the groups (F (4, 35) = 4.139, P > 0.05). It seems that ALNP impairs short-term spatial memory, while quercetin at a dose of 100 mg/kg mitigates this effect.

### Quercetin mitigates the effect of ALNP on the volumes of the CA1, dentate gyrus sub-regions and entire hippocampus 

**Figure 7:**
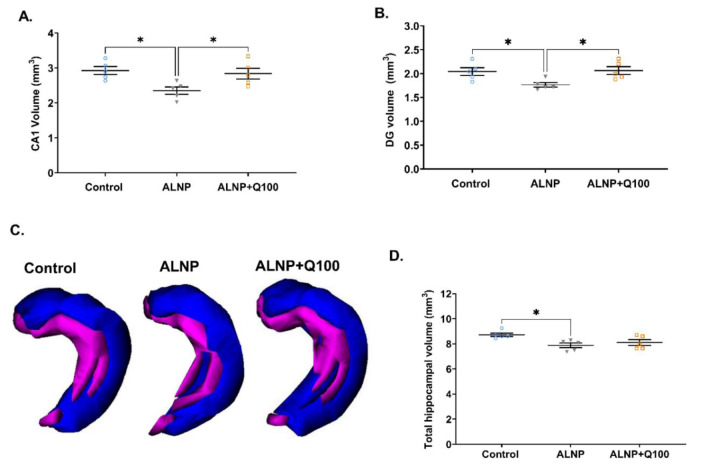
Figure 7. The effects of ALNP with or without quercetin on the volume of cornus ammonium1 (CA1), dentate gyrus (DG) and entire hippocampal regions. The scatter dot plots illustrate the volumes of CA1 (A), DG (B), and the total hippocampus (D) for each group. Three-dimensional reconstructions (3DR) (C) of the CA [blue] and DG [purple]. Each dot represents an individual animal, and the horizontal bars indicate the mean values for each parameter (n = 5 per group). *P < 0.05 indicate statistically significant differences between groups. The groups include: control (saline + CMC 2%), ALNP (aluminum nanoparticles 10 mg/kg + CMC 2%), and ALNP + Q100 (aluminum nanoparticles 10 mg/kg + quercetin 100 mg/kg).

Figure 7(Fig. 7) illustrates the effects of ALNP, both with and without quercetin, on the volumes of the CA1, DG, and the entire hippocampus. One-way ANOVA results indicated significant differences among the groups in the CA1 sub-region (Fig. 7A(Fig. 7), F (2, 12) = 5.932, P = 0.0162). ALNP treatment resulted in a significant reduction in CA1 volume, with a 19.7% decrease compared to control group. In contrast, quercetin administered at a dosage of 100 mg/kg effectively prevented the decrease in CA1 volume, resulting in a 17% increase compared to the ALNP group. Similarly, a significant difference in DG volume was observed among the groups (Fig. 7B(Fig. 7), F (2, 12) = 5.371, P = 0.0216). ALNP significantly reduced DG volume, showing a 13.7% decline compared to control group, while quercetin at 100 mg/kg significantly mitigated this reduction, leading to a 14.5% increase in DG volume compared to ALNP group. Figure 7C(Fig. 7) illustrates the 3DR of the entire hippocampal CA regions (in blue) and the dentate gyrus (in purple). One-way ANOVA results revealed significant differences among the groups regarding the total volume of the hippocampus (Fig. 7D(Fig. 7), F (2, 12) = 5.222, P = 0.0234). The total volume of the hippocampus decreased by 9.6% in the ALNP group compared to the control group. 

Quercetin administered at a dosage of 100 mg/kg increased the hippocampal volume to a level that was not statistically significant when compared to the control group. These results suggest that a dosage of 100 mg/kg of quercetin mitigates the reduction in hippocampal volume induced by ALNP in mice.

### Quercetin mitigates the effect of ALNP on neural cell numbers of CA1 and DG Regions

**Figure 8:**
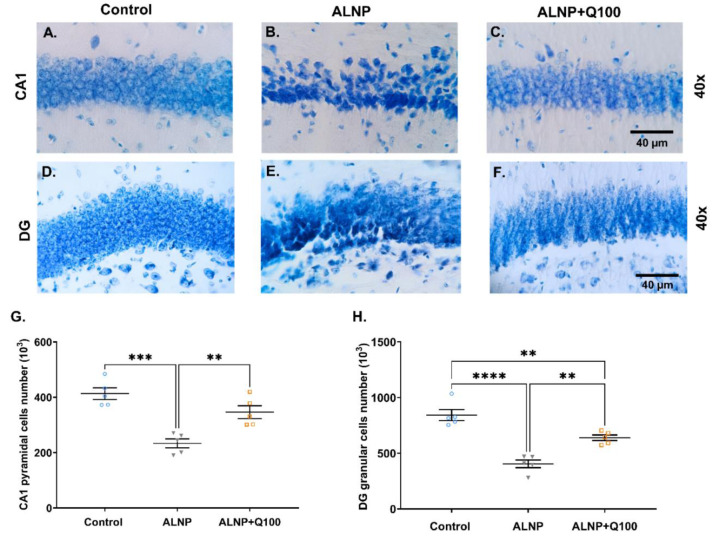
Figure 8. Effects of ALNP with or without quercetin on neural cell numbers in the Cornu ammonis 1 (CA1) and dentate gyrus (DG) regions. Giemsa staining followed by stereological analysis was conducted to evaluate cell numbers in the CA1 and DG regions of the hippocampus. The images illustrate 25 µm sections of the CA1 and DG (A-F). Neurons in the control group exhibited normal morphology (A and D), whereas the ALNP group displayed a reduced neural population characterized by smaller cell sizes, as well as the presence of dead, shrunken, and pyknotic neurons (B and E). In ALNP+Q100, the morphological changes associated with ALNP treatment are mitigated (C and F). The scatter dot plots of the neural cell numbers of the CA1 and DG are shown in Fig 8G and H respectively. Each dot represents an individual animal, and the horizontal bars indicate the mean values for each parameter (n = 5 per group). Statistical significance is denoted as **P < 0.01, ***P < 0.001, and ****P < 0.0001. Scale bar = 40 µm, magnification 40X. The experimental groups include saline + CMC 2% (Control), Aluminum nanoparticle 10 + CMC 2% (ALNP), and Aluminum nanoparticle 10 + Quercetin 100 (ALNP + Q100).

Figure 8(Fig. 8) illustrates the results of neural cells counts of CA1 (pyramidal) and DG (granular) regions of the hippocampus. As depicted in Figures 8A and 8D(Fig. 8), normal neural cells exhibit a uniform blue coloration following Giemsa staining. In contrast, the ALNP-treated group (Figures 8B and 8E(Fig. 8)) displays hyperchromatic and shrunken neural cells, with a reduction in both the number and volume of these cells, accompanied by an increase in intercellular space, indicating neural cell death in both the CA1 and DG sub-regions. However, as shown in Figures 8C and 8F(Fig. 8), the morphological changes associated with ALNP treatment are mitigated when quercetin is administered at a dosage of 100 mg/kg.

The results of the one-way ANOVA indicated significant differences between groups in the total number of pyramidal cells in the CA1 region (Fig. 8G(Fig. 8), F (2, 12) = 20.07, P = 0.0001). The total number of pyramidal cells in the CA1 region of the ALNP group decreased significantly, showing a reduction of 16.22% compared to the control group (P < 0.001). Conversely, the Q100 + ALNP group exhibited a 32.58% recovery in pyramidal cell numbers compared to the ALNP group. 

A one-way ANOVA revealed a significant difference in granular cells of DG gyrus (Fig. 8H(Fig. 8), F (2, 12) = 33.60, P < 0.0001). Additionally, the total number of granular cells in the DG region of the ALNP group exhibited a 52.04% decrease compared to the control group (P < 0.0001). Although a significant difference of 20.9% exists between the control and ALNP + Q100 groups, the ALNP + Q100 group exhibited a significant increase in DG granular cells, rising by 36.7% compared to the ALNP group (P < 0.001). It appears that exposure to ALNP reduces the number of neural cells in the CA1 and DG regions of the hippocampus, while quercetin alleviates these reductions.

### The correlation between the volumes of CA1 and DG areas in relation to their respective neural cell counts

**Figure 9:**
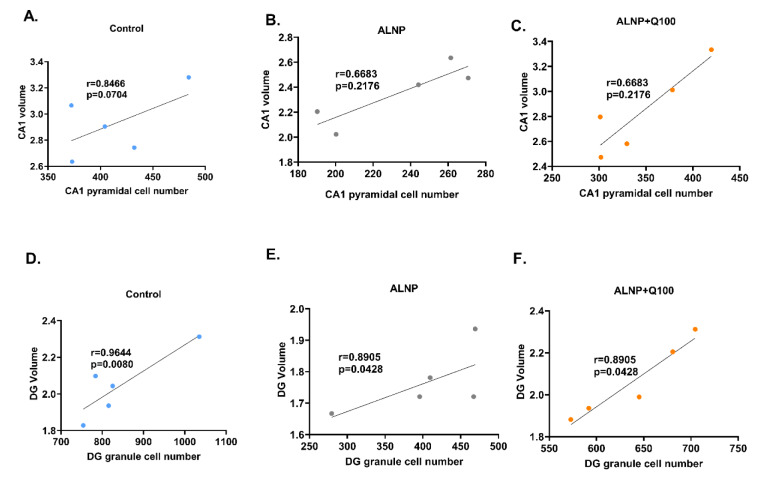
Figure 9. The correlation between the volumes of Cornu ammonis 1 (CA1) and the dentate gyrus (DG) areas in relation to their respective neural cell counts. Correlation analyses were conducted between the number of CA1 pyramidal cells and the CA1 volume in the control (A), ALNP (B), and ALNP+Q100 (C) groups. Additionally, correlation analyses were performed between the number of DG granular cells and the volume of DG in the control (D), ALNP (E), and ALNP+Q100 (F) groups. The parameters were estimated using Pearson's correlation coefficient (r), which represents the correlation coefficient. The experimental groups include saline + CMC 2% (Control), Aluminum nanoparticle 10 + CMC 2% (ALNP), and Aluminum nanoparticle 10 + Quercetin 100 (ALNP + Q100).

To evaluate the potential associations between the reduction in volumes of the CA1 and DG areas and the corresponding decrease in neural cell numbers, Pearson's correlation analysis was employed. The results are illustrated in Figures 9A-F(Fig. 9). No significant association was observed between the number of CA1 pyramidal cells and CA1 volume in any of the groups (Figures 9A-C(Fig. 9)). However, the number of granular cells exhibited a significant correlation with the volume of the DG across all groups. This suggests that the loss of granular cells in the DG area is associated with the reduced volume of this sub-region.

### Quercetin influences hippocampal caspase-3 and MAPKs signaling in ALNP-treated mice

**Figure 10:**
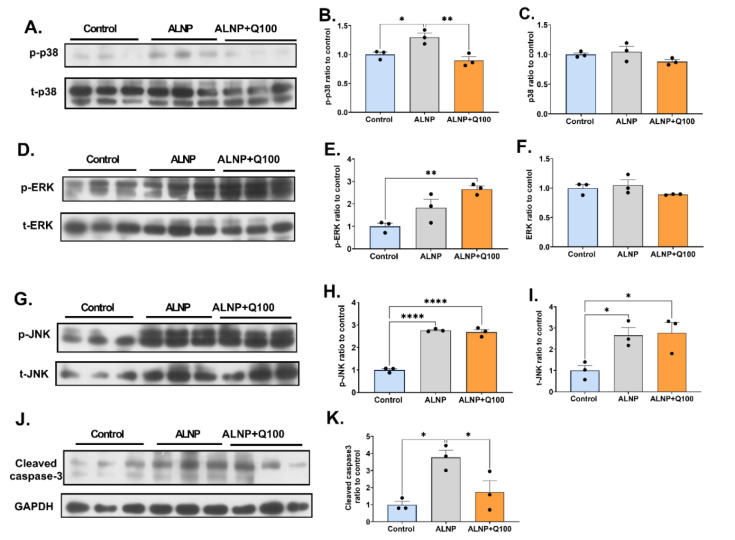
Figure 10. The immunoblotting results of hippocampal MAPKs signaling (A, D, G), cleaved caspase-3 and GAPDH (J) following ALNP treatment, whether administered alone or with quercetin, are presented. Quantitative analyses of p-p38 (B) and t-p38 (C), p-ERK (E) and t-ERK (F), p-JNK (H), t-JNK (I), and cleaved caspase-3 (K) are illustrated. The data are presented as mean ± SEM (n = 3, with subjects randomly assigned to each group). *P < 0.05, **P < 0.01, and ****P < 0.0001 show statistical difference between groups. control (saline + CMC 2%), ALNP (aluminum nanoparticles 10 mg/kg + CMC 2%), and ALNP + Q100 (aluminum nanoparticles 10 mg/kg + quercetin 100 mg/kg).

Western blot analysis revealed significant alterations in hippocampal MAPK signaling and cleaved caspase-3 levels following ALNP treatment, both alone and in combination with quercetin are presented in Figure 10(Fig. 10). One-way ANOVA results demonstrated significant differences among groups in the p-p38 (F (2,6) = 12.11, P = 0.0078), p-ERK (F (2,6) = 11.76, P = 0.0084), p-JNK (F (2,6) = 163.6, P < 0.0001), total JNK (t-JNK, F (2,6) = 7.120, P = 0.0260) and caspase-3 (F (2,6) = 9.534, P = 0.0137) between groups. ALNP at a dosage of 10 mg/kg significantly increased the levels of both p-p38 and caspase-3 (P < 0.05). In contrast, treatment with quercetin at 100 mg/kg significantly restored p-p38 and caspase-3 (P < 0.01 and P < 0.05, respectively). No significant difference was found in total p38 levels (t-p38) across groups. Quercetin treatment at a dosage of 100 mg/kg significantly increased p-ERK levels compared to the control (P < 0.01), while t-ERK levels show no significant differences among the groups. Both ALNP and ALNP+Q100 groups exhibited significant increases in p-JNK (P < 0.0001) and t-JNK (P < 0.05) levels compared to control in the hippocampus. 

## Discussion

In the present study, quercetin alleviated memory deficits induced by ALNP in both the Novel Object Recognition (NOR) and Y-maze spatial memory tasks. The findings of this study confirm recent investigations into the neurotoxic effects of ALNP. For instance, Sun et al. reported that a single bilateral hippocampal infusion of ALNP at doses of 10 or 20 μg/kg in rats resulted in memory impairment and histological alterations in the hippocampus (Sun et al., 2022[65]).

Furthermore, recent studies indicate that a short-term oral treatment with ALNP (10 mg/kg) for 5 days in mice is associated with cognitive deficits (Esmaili et al., 2022[19]; Izadi et al., 2024[29]; Izadi et al., 2023[30]).

The doses and treatment durations of ALNP required to induce memory deficits are significantly lower than those of Al itself. For example, Al should be administered at the dose of 100 mg/kg for 60 days to induce cognitive impairment (Firdaus et al., 2022[22]; Zhao et al., 2020[72]). This difference may be attributed to the smaller size of ALNP compared to bulk Al, which allows it to penetrate the blood-brain barrier (BBB) more easily (Mirshafa et al., 2018[45]). Our findings are in agreement with Shah et al.(Shah et al., 2015[60]), who demonstrated that nanoscale alumina could induce oxidative stress and accelerate Aβ accumulation in the mouse brain, further supporting the link between Al nanoparticles and AD-related neurotoxicity. The current study demonstrates that quercetin, at doses of 10 mg/kg and 100 mg/kg, prevents memory deficits induced by ALNP exposure in the NOR task. In the assessment of spatial reference memory using the Y-maze task, the 100 mg/kg dose of quercetin (but not the 10 mg/kg dose) effectively mitigated the impairing effects of ALNP. Given that the Y-maze evaluates short-term memory, it can be concluded that higher doses of quercetin may be necessary to alleviate short-term memory impairments. Therefore, it appears that quercetin, when administered at an appropriate dose, can prevent the disruption of hippocampal function induced by ALNP. Alternatively, it is plausible that spatial reference memory, as assessed using the Y-maze, is differentially affected by quercetin compared to the NOR task, which assesses object recognition. As the dose of 100 mg/kg of quercetin proved effective in both types of memory tasks, this dosage was chosen for further stereological and molecular assessments. Although quercetin has relatively low oral bioavailability due to its limited solubility and extensive metabolism, oral administration remains the most practical and clinically relevant route for long-term use. Importantly, several studies have shown that its metabolites are detectable in the brain tissue following oral administration (Andres et al., 2018[4]; Dajas et al., 2015[16]; Guo and Bruno, 2015[24]). The neuroprotective effect of quercetin aligns with previous studies demonstrating the cognitive-enhancing effects of quercetin in various animal models of neurological disorders (Nakagawa and Ohta, 2019[48]; Sabogal-Guáqueta et al., 2015[59]). Patil et al. reported that the administration of quercetin (25-100 mg/kg) over a period of 7 days prevented cognitive impairment and reduced oxidative stress in a lipopolysaccharide (LPS)-induced AD model in mice (Patil et al., 2003[50]). Treatment with quercetin (500 mg/kg/day, administered orally for 10 days) in 5XFAD transgenic mice resulted in increased brain Apolipoprotein E (Apo-E) levels and a reduction in insoluble Aβ levels (Zhang et al., 2016[71]). In APP/PS1 transgenic AD mice, intravenous administration of quercetin nanoparticles at doses of 10 to 30 mg/kg for 30 days led to improvements in memory as assessed by the Morris water maze and NOR tasks (Sun et al., 2016[63]). In human studies, a 24-week clinical trial study on healthy Japanese individuals aged 60 to 79 found that quercetin-rich onions may reduce age-related cognitive decline (Nishihira et al., 2021[49]). However, in a randomized, placebo-controlled study involving a large healthy population sample for 12 weeks, quercetin supplementation had no significant effects on cognitive function (Broman-Fulks et al., 2012[12]). In AL induced neurotoxicity, quercetin had been shown to prevent cognitive impairments, cholinergic dysfunction, and oxidative damage in rats (Sharma et al., 2013[61]). The present study, for the first time evaluated the neuroprotective effect of quercetin in ALNP model of memory deficit. As mentioned previously, these findings are significant because the nanoscale size of AL has a greater potential for neurotoxic effects compared to AL in its bulk form. The results of the EPM task revealed that ALNP had no significant effect on the animals' anxiety-like behaviors and general activity, which could be considered interfering factors in memory.

The present study demonstrates that exposure to ALNP results in a significant reduction in the volumes of the CA1 and DG sub-regions, as well as the entire volume of the hippocampus. Furthermore, ALNP administration decreased the total number of pyramidal cells in the CA1 region and granular cells in the DG. These stereological alterations in the hippocampus, which may contribute to the observed memory deficits, are consistent with recent findings (Esmaili et al., 2022[19]). The toxic effects of ALNP on the hippocampal structure observed in this study are consistent with previous reports indicating that AL accumulation in the hippocampus can lead to histological changes, including disorganized cortical layers, neuronal death, pyknosis, and a reduction in cell body density (Abdelhameed et al., 2023[2]; Bittencourt et al., 2022[11]). Furthermore, Atia et al. reported that ALNP treatment induced dystrophic changes in the hippocampus, characterized by small cell degeneration and shrinkage, as well as large pyramidal cells with pycnotic and condensed nuclei in the CA1 and CA3 regions. Additionally, the cytoplasm of some pyramidal cells exhibited significant vacuolation (Atia and Alghriany, 2021[7]). The mechanisms by which ALNP induces these structural changes may involve oxidative stress, neuroinflammation, and the dysregulation of signaling pathways, such as the MAPKs cascade, which are known to play critical roles in regulating neuronal function and survival (Li et al., 2009[39]; Mirshafa et al., 2018[45]; Rai et al., 2024[54]; Xue et al., 2024[67]). The current study demonstrates that quercetin, administered at a dose of 100 mg/kg, effectively mitigates the reductions in the volumes of the CA1 and DG subregions, as well as the total hippocampal volume, induced by ALNP. Furthermore, quercetin at this dosage was able to attenuate the ALNP-mediated decrease in the number of pyramidal cells in the CA1 region and granular cells in the DG. These findings suggest that quercetin may protect against ALNP-induced memory impairment by preserving hippocampal stereological parameters. Quercetin treatment has been shown to mitigate brain histopathological alterations induced by sodium arsenate (Nageshwar et al., 2019[47]) or lead exposure (Musa Omoyine et al., 2021[46]) in rats. Within the hippocampus, quercetin was found to prevent histological changes induced by hypoxia in rats (Prasad et al., 2013[53]). The present study employed stereological techniques, which offer several advantages over traditional histological studies. While histological assessments can be biased due to the selection of slides, inconsistent section thickness, non-random sampling, and inherent variability in neural profiles, systematic random serial sampling provides high accuracy in determining tissue volume and cell numbers and thereby facilitates reliable comparisons between research groups and laboratory findings (Brown, 2017[13]). Significant correlations were found between DG volume and granular cell count across all groups, which is consistent with previous findings (Esmaili et al., 2022[19]). It can be concluded that a reduction in granular neural cells may lead to DG shrinkage. 

The current study revealed that a 10-day treatment course of ALNP activated p38 and JNK signaling within the hippocampus, while having no significant effect on ERK signaling. Previous research demonstrated that a 5-day oral ALNP treatment led to the activation of hippocampal p38 and ERK, with no significant effect on JNK (Mehrbeheshti et al., 2022[44]). These discrepancies may be attributed to variations in protocols, such as the route of administration (which may affect ALNP absorption) or differences in treatment duration. 

Quercetin, in contrast, mitigated the increased activation of p38 induced by ALNP. Additionally, it enhanced the activated (phosphorylated) form of ERK within the hippocampus. P38 activation is believed to contribute to the pathological events associated with human AD (Asih et al., 2020[5]; Lee and Kim, 2017[38]). It has been suggested that p38 phosphorylation is positively regulated by ERK and negatively modulated by JNK (Zaplatic et al., 2019[69]). Previously, the protective effect of quercetin was found to be associated with ERK over-activation (Zaplatic et al., 2019[69]). Therefore, it is plausible that ERK over-activation in the present study diminishes p38 phosphorylation induced by ALNP.

The results of the current study indicate that ALNP increases caspase-3 cleavage within the hippocampus. This finding aligns with recent studies (Esmaili et al., 2021[20]; Mehrbeheshti et al., 2022[44]). Caspase-3 cleavage is recognized as a hallmark of apoptotic cell death (Falcicchia et al., 2020[21]; Sun and Nan, 2017[64]). This result is consistent with the aforementioned stereological evaluations, which demonstrate increased neural cell death in the CA1 and DG sub-regions of the hippocampus. Conversely, quercetin reduces the cleavage of caspase-3 induced by ALNP. Given the proposed activating effect of p38 MAPKs on caspase-3 cleavage, the modulatory effect of quercetin on hippocampal p38 signaling may contribute to its neuroprotective properties (Chen et al., 2020[14]). These findings are consistent with earlier studies that emphasize the anti-apoptotic effects of quercetin on neural cells (Chen et al., 2021[15]; Du et al., 2018[18]).

In conclusion, the present study demonstrated that quercetin improved memory performance in a mouse model of Alzheimer's disease (AD) induced by aluminum oxide nanoparticles (ALNP). The memory-enhancing effects of quercetin were associated with the restoration of hippocampal structure, including volume and neuronal cell number, as well as modulation of key signaling pathways such as ERK, p38, and caspase-3. Importantly, given that ALNP are largely insoluble under physiological conditions, the observed neurotoxic effects are unlikely to result from free Al³⁺ ions but rather from nanoparticle-specific mechanisms- such as oxidative stress, mitochondrial dysfunction, and signaling pathway disruption. These findings suggest that ALNP may exert distinct neurotoxic effects compared to conventional ionic Al compounds like AlCl₃. Therefore, quercetin may be considered a promising candidate for mitigating cognitive dysfunction caused by nanoparticulate forms of Al exposure.

## Notes

Mohammad Shabani and Maryam Moosavi (Nanomedicine and Nanobiology Research Center, Shiraz University of Medical Sciences, Shiraz, Iran; Tel: +98 713 6122284, Email: maryammoosavi@sums.ac.ir) contributed equally as corresponding author.

## Declaration

### Acknowledgements

This work was extracted from a thesis written by Zahra Esmaili and was supported by a grant (No. 401000738) from Kerman University of Medical Sciences, Kerman, Iran. 

### Funding Declaration

This work was supported by a grant (No. 401000738) from Kerman University of Medical Sciences, Kerman, Iran. 

### Conflict of Interest

The authors declare that they have no conflict of interests.

### Ethical statement

The protocols were approved by the Ethics Committee for Scientific Study at Kerman University of Medical Sciences (license # IR. KMU.AH.REC.1402.046). The present article does not contain any experiment on human participants performed by any of the authors.

## Figures and Tables

**Table 1:**
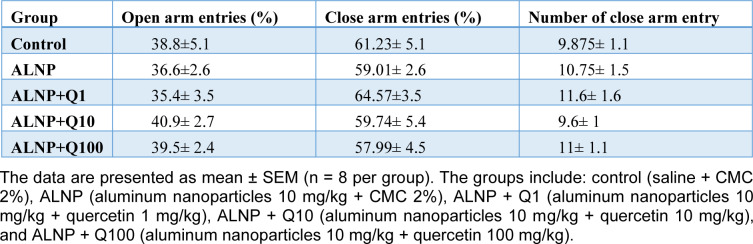
The effect of aluminum nanoparticle (ALNP) with and without quercetin (Q) on anxiety-like behaviors measured using the elevated plus maze (EPM)

**Figure 1:**
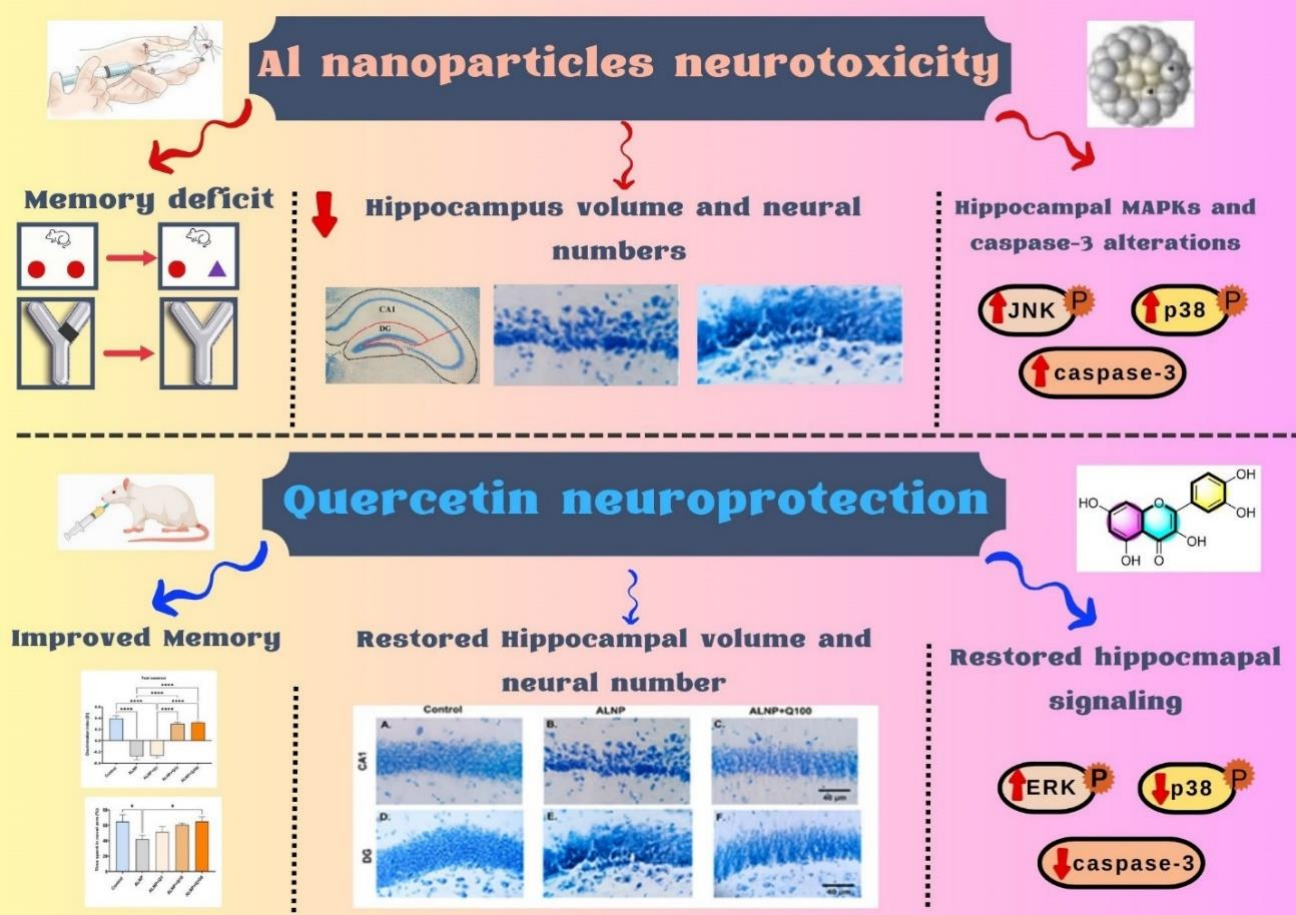
Graphical abstract
